# The ECMO PK Project: an incremental research approach to advance understanding of the pharmacokinetic alterations and improve patient outcomes during extracorporeal membrane oxygenation

**DOI:** 10.1186/1471-2253-13-7

**Published:** 2013-03-21

**Authors:** Kiran Shekar, Jason A Roberts, Maree T Smith, Yoke L Fung, John F Fraser

**Affiliations:** 1 Critical Care Research Group, Adult Intensive Care Services, The Prince Charles, Hospital and The University of Queensland, Brisbane, QLD, 4032, Australia; 2 Burns Trauma and Critical Care Research Centre, The University of Queensland, Brisbane, QLD, Australia; 3 Centre for Integrated Preclinical Drug Development, The University of Queensland, Brisbane, QLD, Australia; 4 Critical Care Research Group, The Prince Charles Hospital and The University of Queensland, Brisbane, QLD, Australia

**Keywords:** Pharmacokinetics, Extracorporeal membrane oxygenation, Pharmacodynamics, Therapeutic failure, Toxicity

## Abstract

**Background:**

Extracorporeal membrane oxygenation (ECMO) is a supportive therapy and its success depends on optimal drug therapy along with other supportive care. Emerging evidence suggests significant interactions between the drug and the device resulting in altered pharmacokinetics (PK) of vital drugs which may be further complicated by the PK changes that occur in the context of critical illness. Such PK alterations are complex and challenging to investigate in critically ill patients on ECMO and necessitate mechanistic research. The aim of this project is to investigate each of circuit, drug and critical illness factors that affect drug PK during ECMO.

**Methods/design:**

An incremental research plan that encompasses *ex vivo* experiments for drug stability testing in fresh human and ovine whole blood, *ex vivo* drug disposition studies in standard and modified adult ECMO circuits primed with fresh human or ovine whole blood, PK studies in healthy and critically ill ovine models of ECMO with appropriate non ECMO controls and an international mutli-centre clinical population PK study will be utilised to comprehensively define the PK alterations that occur in the presence of ECMO. Novel drug assays that will allow quantification of multiple drugs in small volumes of plasma will also be developed. Mixed-effects regression models will be used to estimate the drug loss over time in *ex vivo* studies. Data from animal and clinical studies will be analysed using non-linear mixed-effects models. This will lead to generation of PK data that enables the development evidence based guidelines for antibiotic, sedative and analgesic drug therapy during ECMO.

**Discussion:**

Systematic research that integrates both mechanistic and clinical research is desirable when investigating the complex area of pharmacokinetic alterations during ECMO. The above research approach will provide an advanced mechanistic understanding of PK during ECMO. The clinical study when complete will result in development robust guidelines for prescription of 18 commonly used antibiotic, sedative and analgesic drugs used in ECMO patients. This research may also pave the way for further refinements in circuitry, drug chemistry and drug prescriptions during ECMO.

**Trial registration:**

ACTRN12612000559819.

## Background

Extracorporeal membrane oxygenation (ECMO) temporarily supports patients with severe cardio-respiratory failure that is not responsive to maximal conventional treatment [1-4]. Following the 2009 H1N1 pandemic, ECMO has re-emerged as a versatile device that not only provides cardio-respiratory support when medical therapy fails but also compliments existing mechanical cardiopulmonary assist devices, heart /lung transplantation, cardiology and hospital based cardiopulmonary resuscitation services effectively. As ECMO is a supportive therapy, effective drug therapy directed at reversing the underlying disease is critical to ensure successful liberation from ECMO. Indeed, the clinicians applying ECMO recognise that contemporary use of this therapy is far from perfect with patients suffering ongoing morbidity because the clinicians are no longer able to confidently achieve the desired effects from pharmacotherapies. Published data demonstrates that ECMO dramatically affects pharmacokinetics (PK) in the most severely ill patients who already have significant PK changes [5-8].

**Figure 1:**
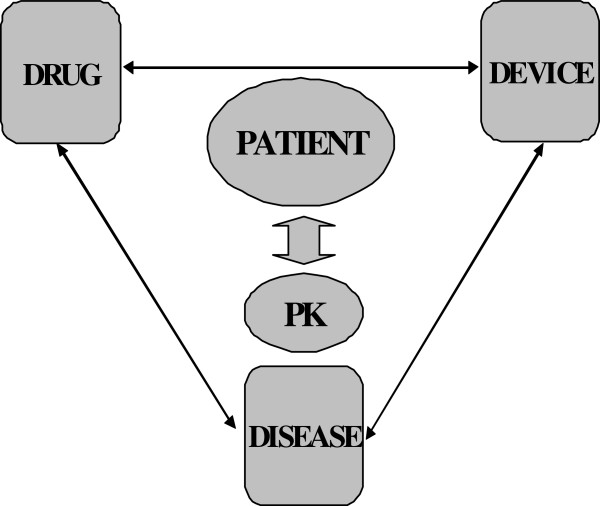
Three D’s affecting pharmacokinetics during ECMO.

It is essential that each of the drug, device and disease factors affecting PK during ECMO (Figure 1) is studied to improve treatment and outcomes of patients. We hypothesise that ECMO negatively alters the PK of sedative, analgesic and antibiotic drugs and their metabolites independent of patient and pathological factors, thereby contributing to elevated risk of therapeutic failure, drug toxicity and/or an emergence of microbial resistance in critically ill patients receiving ECMO. Our aim is to use an incremental research approach that include studies investigating drug, circuit and critical illness factors in isolation and combined to arrive at meaningful conclusions.

### ECMO circuits are not passive conduits for blood

**Figure 2:**
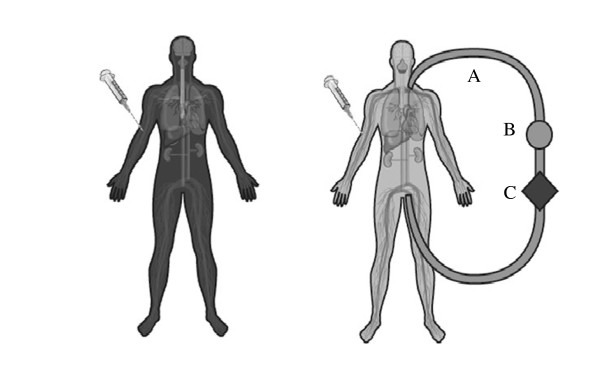
**Significant sequestration of drugs in the ECMO circuit increases their volumes of distribution leading to suboptimal drug concentrations in the body.** A mere increase in administered dose for all drugs during ECMO may not suffice, as the less sequestered drugs may reach toxic levels. **A** - PVC tubing, **B**- pump, **C**- oxygenator (Reproduced with Permission, Shekar et al Journal of Crit Care 2012).

In critically ill patients not receiving ECMO, it has been shown PK changes can result in highly significant changes to drug exposure through interactions between the patient, pathology and the drug [9-12]. The ECMO system introduces additional variables, which are the circuit itself, and the effects of systemic inflammation due to the prolonged use of an extracorporeal circuit. Sequestration of drugs in the circuit, increased volume of distribution (Vd) and decreased clearance (CL) are the major PK changes associated with ECMO [8], although the extent of change remains poorly characterised. Published data from neonatal circuit studies highlight the influence that drug properties such as molecular size, degree of ionization at physiological pH, lipophilicity and plasma protein binding have on drug disposition during ECMO [13,14]. In a manner analogous to the lung it mimics, ECMO is critically dependent upon the large surface area of the oxygenator and associated tubing to ensure adequate blood flows through the circuit and facilitate gas transfer. This bio-synthetic interface results in significant sequestration of the administered drugs resulting in a compartmental effect on PK (Figure 2). The type and age of circuit components including type of the pump, oxygenator and tubing as well as circuit priming may influence the level of drug sequestration [15-18]. Patient factors such as systemic inflammation, haemodilution, bleeding and transfusion, organ dysfunction and renal replacement therapy all add to the clinical challenges of drug dosing during ECMO [8].

### The burden of altered pharmacokinetics during ECMO

There is increasing awareness of the implications of altered PK during critical illness in adult patients [19-21]. The PK changes during critical illness appear to be magnified during ECMO. This can affect any drug, however given the scientific and clinical PK gap, robust PK data for sedative, analgesic and antibiotic drugs are urgently required.

#### Excessive sedation use and related morbidity

Sedation practices in the ICU are changing and emerging data supports its judicious use [22,23]. Neonatal studies consistently demonstrate a need to escalate sedative doses during ECMO [13,24-26]. In a retrospective review of 30 patients [7], the average 24-hourly dose increased by 18 mg per day for midazolam (95% CI: 8, 29 mg, p=0.001) and 29 mg per day for morphine (95% CI: 4, 53 mg, p=0.021) from the first day of ECMO. The VV group required a daily midazolam dose that was 157 mg higher on average than the VA group (95% CI: 53, 261 mg, p=0.005). Patients often received up to 1500 mg of morphine and midazolam per day despite supplemental sedation with propofol, dexmedetomidine and thiopentone. By acting as a reservoir, ECMO may also prolong the pharmacological effect of sedatives even after drugs have been ceased. This is concerning as it is now well established that excessive sedation in critically ill patients is associated with increased mortality and morbidity [27].

#### Infection, antibiotic failure, drug toxicities and emergence of microbial resistance

ECMO is a supportive therapy and not a disease modifying treatment in itself. The success of ECMO, especially in patients with severe pneumonia or a pandemic viral respiratory illness relies heavily on the success of antiviral/antibiotic therapy. Optimal antibiotic therapy in these patients is a balance between potency and exposure [12,28-31]. A recent review of Extracorporeal Life Support Organization (ELSO) data [32] revealed a total of 2,418 infections during 20,741 (12%) ECMO cases. Infections increased the duration of ECMO, post-ECMO ventilator support and were associated with an increased risk of death. Neonatal studies have reported severe PK variations, however limited data is available to guide antibiotic therapy in adults [13,16,33-37]. Sub-optimal prescription of antibiotics in patients on ECMO can worsen the problem by selecting for resistant microorganisms [29].

## Methods/design

### A rational approach to understand the pharmacokinetic changes

**Figure 3:**
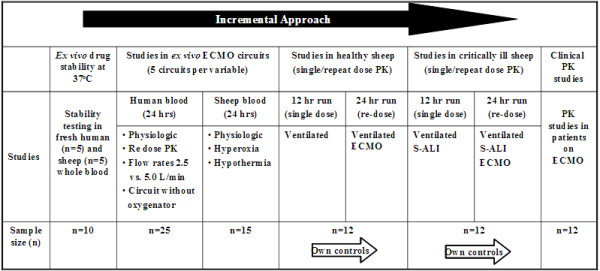
**Proposed approach to quantify the PK changes and identify factors underlying these changes to inform the development of guidelines for antibiotic and sedative drug therapy during ECMO.** S-SLI-smoke inhalation acute lung injury.

**Figure 4:**
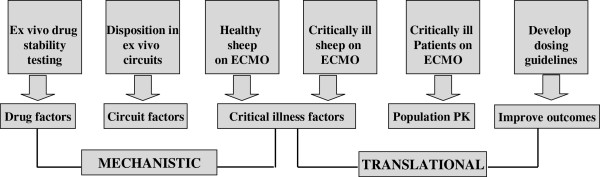
Proposed methods to understand the pharmacokinetic changes and optimise drug therapy during extracorporeal membrane oxygenation (ECMO).

Although data from clinical studies of the impact of ECMO on altering the PK of drugs used in patients on ECMO will have great applicability for optimisation of pharmacotherapy, mechanistic research is required to identify the specific factors contributing to these PK changes. To gain insight into these factors, research using simulated circuits and large animal models are required so that individual variables can be altered in a systematic manner enabling the impact of each change to be quantified in an accurate and cost-effective manner. Additionally, this will define the interplay between critical illness and the extracorporeal circuit that result in altered PK during ECMO. The new knowledge to be generated has major implications for improving patient outcomes during ECMO therapy and extracorporeal technology in general. A proposed research plan that is being currently being implemented uses an incremental approach as shown schematically in and Figures 3 and 4. The study drugs are tabulated in Table 1.

**Table 1:** Study drugs for which mechanistic and clinical pharmacokinetic (PK) data will be generated

**Studies**	**Sedatives & analgesics**	**Antivirals /antifungals**	**Antibacterials**
Ex vivo stability	Morphine	Fluconazole	Ceftriaxone
Ex vivo circuits	Morphine -3 -glucuronide	Caspofungin	Meropenem
Ovine ECMO	Morphine -6 –glucuronide		Vancomycin
Population PK	Fentanyl, nor-fentanyl		Ciprofloxacin
	Midazolam		Gentamicin*
	1 & 4 hydroxy midazolam		
Ex vivo stability	Propofol ,Thiopentone	Oseltamivir	Piperacillin/tazobactum Ticarcillin/clavulunate
Population PK	Dexmedetomidine	Voriconazole	Cefepime, Linezolid

OC –oseltamivir carboxylate. * Gentamicin will not included in the *ex vivo* circuit studies due to its incompatibility with other study drugs.

### Drug factors

*Ex vivo* controls to examine baseline stability of drugs at 37°C is an important consideration in interpreting the PK alterations during ECMO. Stability testing in fresh human and sheep whole blood will be performed for all study drugs (Table 1). This is critical as drug losses in the circuit can only be meaningfully interpreted after establishing stability. Preliminary results highlight this as drugs such as meropenem [38] are highly unstable at 37°C.

### Circuit factors

These studies will identify the PK changes attributable to the circuit and drugs and will be used to describe single and repeat dose kinetics in standard circuits.

#### Disposition of drugs in standard ECMO circuit

**Figure 5:**
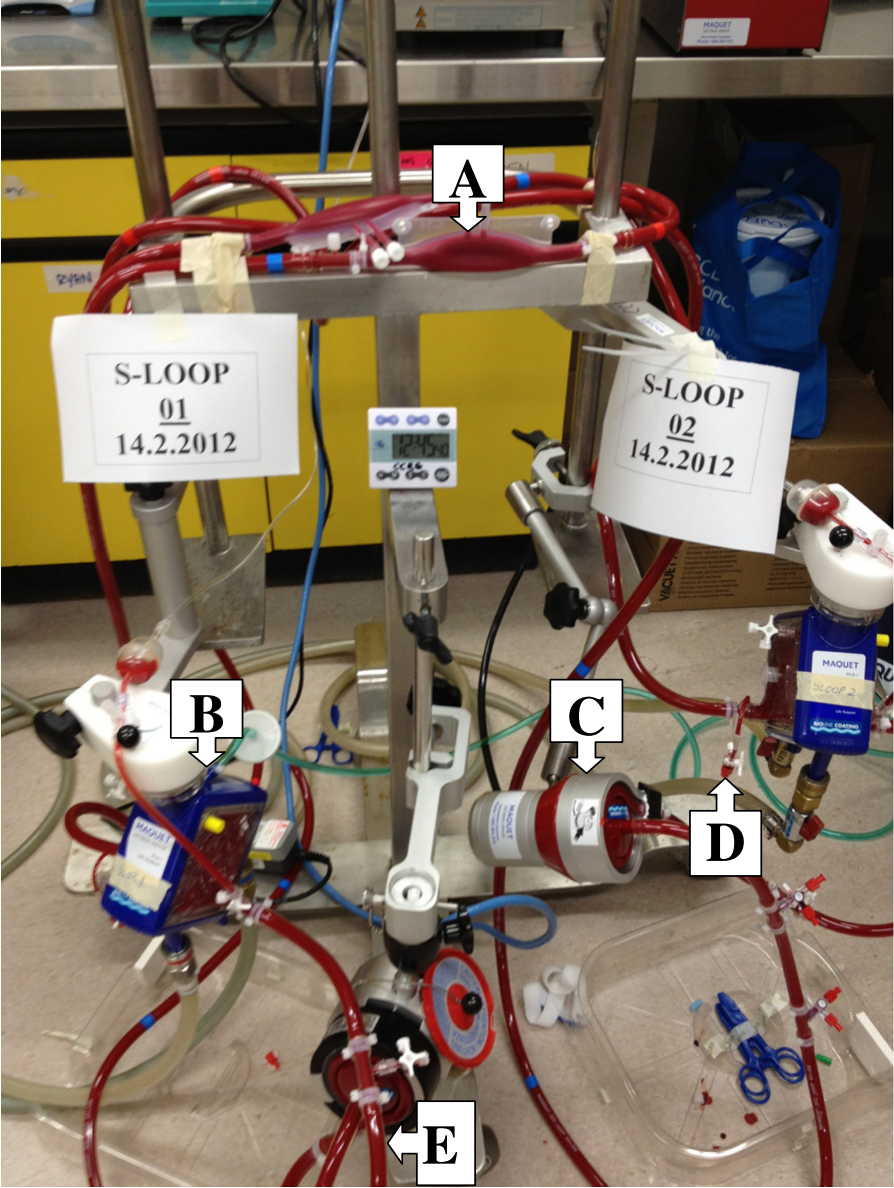
***Ex vivo *ECMO circuit model. A** -reservoir bladder, **B**- oxygenator, **C**- centrifugal pump, **D**-drug injection and sampling port, **E**- circuit tubing.

A validated *ex vivo* model of ECMO has been previously published [38,39]. Briefly, Maquet PLS ECMO circuits will be used (Maquet Cardiopulmonary AG, Hechinger Straße, Germany). A reservoir bladder (Medtronic R38) will allow sampling from the closed circuit (Figure 5). The circuit will be primed with Plasmalyte, 4% albumin followed by fresh whole blood to obtain a post oxygenator pressure of 230–250 mmHg. The final estimated volume of the pressurised circuit is 668 mL. A centrifugal pump maintained a circuit flow rate of 4–5 L /min. Oxygen tension and circuit temperature and pH will be maintained at 100–150 mm Hg and 37°C. Carbon dioxide gas or sodium bicarbonate solution will be added to the circuit to maintain the pH of the circulating blood in the range 7.25–7.55. Study drugs (Table 1) will be injected post oxygenator to achieve clinically relevant concentrations in the circuit. Serial samples will be obtained post oxygenator over 24 hours. For re-dose PK studies, study drugs will be reinjected at 6, 8 or 12 hours (as per clinical dosing guidelines). This will further investigate potential saturation of the circuit with time and its affect on drug disposition during ECMO.

### Disposition of drugs in modified ECMO circuit

#### Circuit primed with fresh whole human Blood

##### Circuit without oxygenator

These studies will examine the role of the oxygenator in sequestering drugs in the ECMO circuit. By comparing the data from standard ECMO circuit experiments, the relative contribution of the oxygenator may be quantified. It has been established in neonatal circuit studies that the type of the pump and the oxygenator can influence drug PK [15].

#### Circuit at varying flow rates (2.5 and 5.0 L/min)

Flow rates are thought to influence PK [40], however this has not been adequately tested. Higher ECMO flows usually reflect greater severity of illness and identifying the PK alterations is essential to maximise the chances of survival in this very unwell subgroup of patients. This experiment will also provide insight into whether or not flow rate adjusted standardisation of drug therapy is required.

### Circuits primed with fresh whole sheep blood

#### Circuit under hyperoxic conditions (PaO2 300–400 mm Hg)

Hyperoxia is not uncommon in patients receiving ECMO [41]. Hyperoxic conditions may affect PK by changes in the catalytic activity of drug metabolising enzymes and changes in membrane permeability, affecting drug distribution [42]. Carbon dioxide gas or sodium bicarbonate solution was added to the circuit to maintain the pH of the circulating blood in the range 7.25–7.55.

#### Circuit under hypothermic conditions (32–34°C)

Hypothermia can affect PK significantly [43,44] however there is limited published data. Circuits will be primed with sheep blood as it is relatively easy to replicate an *in vivo* experiment if required in sheep. The cooling device (Jostra™ Heater-Cooler Unit HCU 30 A) will be added to the ECMO circuit to induce hypothermia. This is relevant as patients on ECMO following CPR often receive therapeutic hypothermia as part of their post resuscitation care. Hypothermia may sometimes be induced to minimise oxygen consumption during VV ECMO. Patients on cardiopulmonary bypass are routinely exposed to hypothermia. Understanding the effect of hypothermia on PK is an important aspect for optimisation of drug dosing during ECMO.

### Host factors

#### Healthy and critically ill controls

Baseline PK samples will be obtained from healthy sheep and sheep with smoke inhalation acute lung injury (S-ALI) over a 12 hour period prior to commencement of ECMO. In an appropriately equipped theatre, a central venous line will be placed in the right internal jugular vein (IJV) under local anaesthesia. Alfaxalone, ketamine and midazolam was used for induction and maintenance of anaesthesia. Buprenorphine 0.01 mg/kg will be used for supplemental analgesia. The sheep will be intubated and ventilated with a Hamilton Galileo ventilator (Hamilton Medical AG). The facial artery will be cannulated for invasive arterial blood pressure monitoring. A pulmonary artery catheter will provide continuous measurements of the central venous pressure, mixed venous saturation and cardiac output (CO). Additional sheaths will be placed in both IJV to facilitate ECMO cannulation and intra-cardiac echo (ICE). Sedative study drug infusions will be titrated to clinical effect. Antibiotics will be infused over 30 mins and serial blood samples will be obtained for drug assays using validated LC-MS/MS methods, and subsequent PK analysis.

For critically ill control sheep, S-ALI will be induced using a validated, reproducible technique that has been published [45]. Briefly, a stainless steel plate will be heated to 750°C and placed on top of 8 g of cotton in a cup. The smoke resulting from combustion will be delivered to the sheep by manual compression of the bellows (tidal volume VT, 10–12 mL/kg) to achieve a carboxyhaemoglobin content of 45–50% is achieved. The sheep will be ventilated using ARDSNet criteria (VT 4–6 ml/kg, PEEP 10–15 cm H_2_O) for lung protective ventilation [46]. Sedative study drug infusions will be titrated to clinical effect. Antibiotics will be infused over 30 mins and serial blood samples will be obtained for drugs assays using validated LC-MS/MS methods, and subsequent PK analysis. Such an approach will provide insights into the effects of critical illness on sedative and antibiotic drug PK.

#### Healthy sheep on ECMO

**Figure 6:**
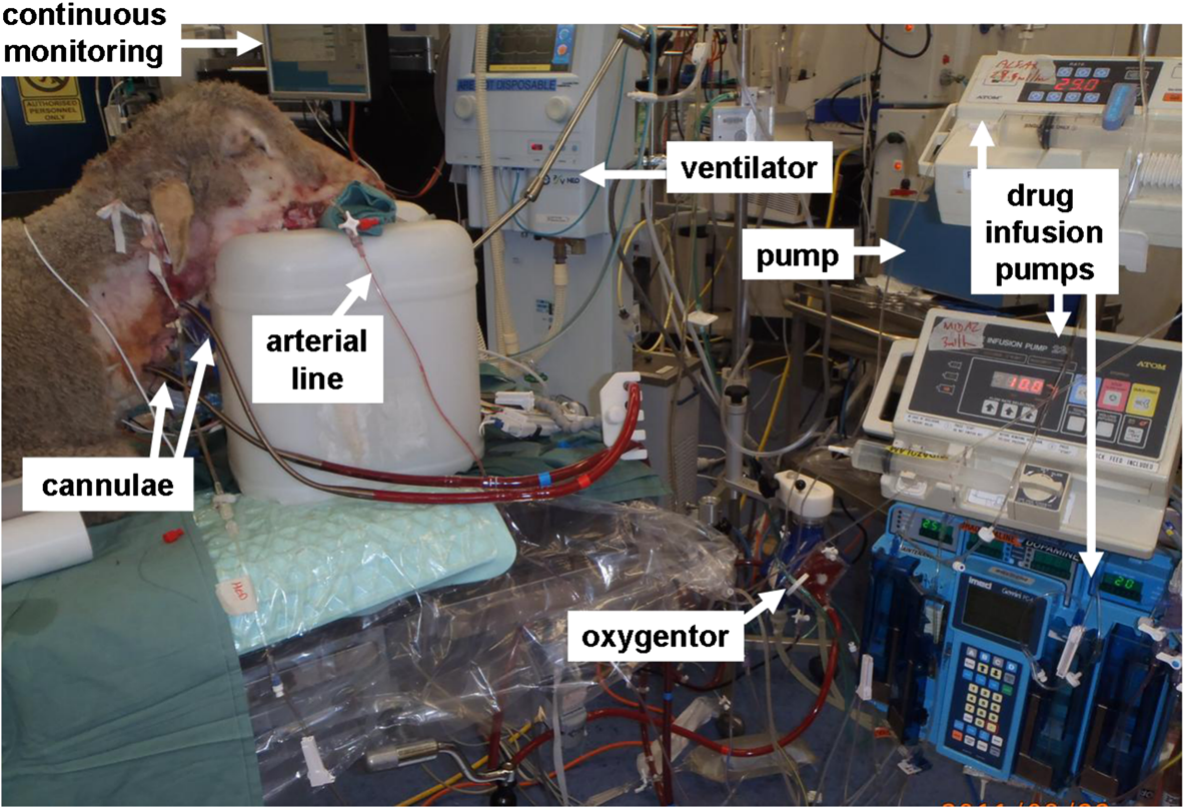
**Validated ovine ECMO model.** Reproduced with permission, Fung et al, ISBT Science Series 2012.

Following 12 hours of ventilation and PK sampling the healthy control sheep will be maintained on ECMO for 24 h. We have recently published a detailed description of our ovine model (Figure 6) of ECMO [39]. Cannulation will be performed in the supine position by rewiring the previously placed IJV venous sheaths. A 21Fr (50 cm) femoral Carmeda Bioactive Surface coated (CBAS®) venous cannula (Medtronic Inc, Minneapolis, MN, USA) will be inserted into the right IJV using a Seldinger technique and positioned using intra cardiac echocardiography (ICE) [47] in the proximal inferior vena cava (IVC). A 19Fr (50 cm) Carmeda coated femoral venous cannula will be used for return blood and also inserted in the right IJV and positioned at the mid right atrium using ICE. Pump speeds will be titrated to target flows at least 2/3^rd^ of pre-ECMO CO (or 60–80 mL/kg). Sedative study drug infusions will be titrated to clinical effect. Antibiotics will be infused over 30 mins upon commencement of ECMO and at 8 and 12 h (for re-dose PK) to obtain serial blood samples for drug assays using validated LC-MS/MS methods, and subsequent PK analysis.

#### Critically ill sheep on ECMO

After 12 h of lung protective ventilation, the control S-ALI sheep will be maintained on ECMO for 24 h. Cannulation, ECMO set up and initiation of ECMO have been described in earlier sections. Sedative study drug infusions will be titrated to clinical effect. Antibiotics will be infused over 30 mins upon commencement of ECMO and at 8 and 12 h (for re-dose PK) to obtain serial blood samples for drugs assays using validated LC-MS/MS methods, and subsequent PK analysis. Upon completion of these studies, PK data from critically ill sheep on ECMO will be compared with data from controls and healthy sheep on ECMO to obtain crucial PK data that will inform our understanding of the factors underpinning the PK changes induced by ECMO that is distinct from the impact of critical illness itself.

The physiologic data collection for the sheep experiments will include; weight, advanced haemodynamic and respiratory monitoring data, ECMO flow rates, urine output, fluid balance, inotrope and vasopressor use and blood loss if any. Eight hour urinary creatinine clearance, serum creatinine, serum total protein, serum albumin, *alpha1-acid glycoprotein*, serum bilirubin, alanine aminotransferase (ALT) measurements will be performed prior to (controls) and during ECMO. Pharmacokinetic studies in critically ill patients on ECMO.

An international multi-centre, clinical PK study [48] will enrol critically ill patients admitted to the intensive care units in Australia and New Zealand. The study centres include; The Prince Charles Hospital, Brisbane, St Vincent’s Hospital, Sydney; The Alfred, Melbourne; Auckland City Hospital, Auckland and Princess Alexandra Hospital, Brisbane. Informed consent will be obtained from the patients or from their next of kin as appropriate. A total of 10–12 patients will be enrolled for each study drug (Table 1) for this descriptive study. Sedative drugs will be titrated to clinical sedation scores and bispectral index. Antibiotic drug selection and dosing is at the discretion of the treating clinicians. In some patients, blood samples relating to only antibiotics may be collected, whereas in other patients, samples for analysis of analgesics and sedatives may also be collected. Patient selection will be based on the below criteria;

#### Inclusion criteria

• Age > 18 years and < 90 years

• Currently undergoing ECMO for respiratory and/or cardiac dysfunction

• Clinical indication for the antibiotics listed in Figure 3

• Clinical indication for the sedatives and analgesics listed in Figure 3

#### Exclusion criteria

• No consent

• Known allergy to study drug

• Pregnancy

• Serum bilurubin > 150 μmol/L

• Ongoing massive blood transfusion requirement (> 50% blood volume transfused in the previous 8 hours)

• Therapeutic plasma exchange in the preceding 24 hours

Our clinical service model of ECMO has been recently published [49]. Feasibility studies are now completed and the study protocol has now been validated and published [48]. Eight hour urine creatinine or effluent creatinine (in patients on renal replacement therapy [RRT]) will provide estimates for renal clearance. Plasma assays and PK modelling will be undertaken for the study drugs (Table 1) using techniques described below.

### PK sample analysis

To reduce the sample burden per patient, validated bioanalytical methods are required to quantify multiple drugs and their metabolites selectively and sensitively in small volumes of plasma. A validated bioanalytical method that uses a fully automated on-line solid phase extraction (SPE) system (Symbiosis, SPARK Holland) combined with liquid chromatography-mass spectrometry (LC-MS/MS –API 5000) to simultaneously quantify morphine, morphine 3-β-D-glucuronide, morphine 6-β-D-glucuronide, midazolam, 1- hydroxymidazolam, 4-hydroxymidazolam, fentanyl and nor-fentanyl in samples of human plasma has been developed [50]. The technique will also be expanded to analyse propofol, thiopentone and dexmedetomidine. This approach enables simultaneous measurement of the plasma concentrations of these molecules of interest with high accuracy and precision in a single specimen. Previously developed and validated antibiotic assays (HPLC and LC-MS/MS) will be used in these studies.

### PK modelling and statistical analysis

The sample size calculations used 10 circuits/subjects, with 10 observations over time per circuit/subject, and an 80% power with a 2-sided 5% significance level. The detectable differences over time are on a standardised scale (Cohen’s d). The within correlations are from previous data. We have the power to detect relatively small changes with our small sample sizes because of the multiple observations per circuit/subject.

#### Circuit studies

Data will be plotted over time and analysed for statistically significant temporal losses. Mixed-effects regression models with random slopes will be used to estimate the loss over time. Octanol-water partition coefficients (log *P*) for the individual drugs are available from the University of Alberta Drug bank website. We will examine the relation between the partition coefficients and the extent of drug loss in the circuit using simple linear regression. Correlation between log *P* values and drug loss will be calculated by using two-sided Spearman test.

#### Animal and clinical studies

Data from these studies will be analysed using non-linear mixed-effects models. This allows the estimation of typical population PK parameters and their inter- and intra-individual variability, plus the estimation of residual random variability. We will fit random intercepts and slopes to allow for between patient differences in their average response and changes over time. This modelling allows us to visualise the average patient and individual patients. It also allows PK to be described in the absence of fixed protocol times, making it ideally suited to calculate PK parameters from drug concentration data collected at with varying times during routine care. Differential equations will be used to describe the population PK of study drugs and their metabolites expressed as PK parameters. Where relevant, results will be normalised to a median patient bodyweight of 70 kg, using allometry.

### Ethical considerations

Appropriate ethics approval has been obtained for all the phases of the ECMO PK project,

• *Ex vivo* circuit experiments using human blood (HREC/12/QPCH/90)

• *In vivo* ovine studies and *ex vivo* circuit experiments that utilise sheep blood (approval no. 1100000053)

• Multi-site ethics approval for the clinical studies in Australia (HREC/11/QPCH/121)

• Single-site ethics approval for the clinical study in New Zealand (LRS/12/06/020)

### Collaborating organisations

This project is co-ordinated by The Critical Care Research Group at The Prince Charles Hospital in Brisbane, Australia. This group will collaborate closely with The Burns Trauma and Critical Care Research Centre, and The Centre for Integrated Preclinical Drug Development, The University of Queensland in Brisbane for antibiotic and sedative drug assays. The Critical Care Research Group will also collaborate closely with all clinical sites involved in the multi-centre population PK study.

## Discussion

**Figure 7:**
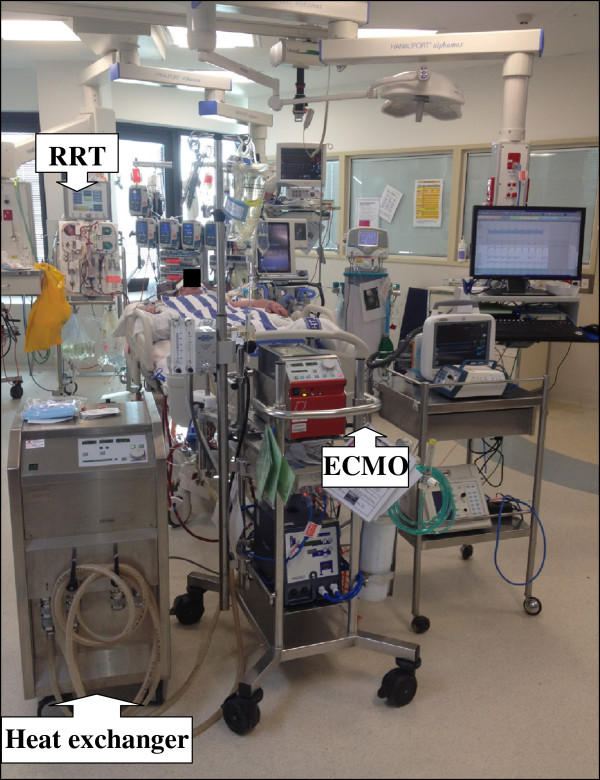
**Challenges in drug dosing during extracorporeal membrane oxygenation (ECMO).** This critically ill patient received concomitant venovenous ECMO, renal replacement therapy (RRT) and induced hypothermia, all of which can significantly alter pharmacokinetics of vital drugs.

This research will not only identify the drugs that are most suitable for use during ECMO but our findings will also inform the development of strategies for drug administration using PK/PD principles in critically ill patients receiving ECMO. These patients receive a variety of pharmacological and other extracorporeal therapies such as RRT and these modalities have a potential to interact with each other (Figure 7). A lack of understanding of the impact of ECMO on drug Vd and CL predisposes to an increased likelihood of therapeutic failure or drug toxicity. PK modelling is crucial to drug safety. The ECMO PK Project seeks to provide the key information for development of evidence-based dosing schedules and sedation protocols for use by clinicians looking after patients receiving ECMO.

Using the right sedative agent at an appropriate dose may minimise ICU morbidity related to risk of infections, duration of mechanical ventilation and length of hospital stay, inotrope and vasopressor requirement, drug withdrawal, post traumatic stress etc. This not only has resource implications but significantly affects patient outcomes [27]. The clinical study will also evaluate the adequacy of existing ICU sedation protocols as compared to bispectral index monitoring and provide data to inform recommendations for improving sedation practices during ECMO.

There is widespread consensus that in-hospital antibiotic resistance influences patient outcome and the allocation of resources. Optimal antibiotic prescription has significant implications not only for the patient on ECMO but also for other ICU patients and the community in general. Antibiotic PK studies in patients receiving ECMO will help the development of dosing regimes that are effective against the microorganism, but not harmful to the patient. The right dose of the right antibiotic will not only lead to improved microbiological and clinical cure rates in an individual patient, but also will reduce the emergence of multi-resistant organisms.

## Conclusions

Systematic research that integrates both mechanistic and clinical research is necessary when investigating the complex area of pharmacokinetic alterations during ECMO. The methods described in this paper will result in an advanced understanding of drug, circuit and critical illness factors that influence PK during ECMO. This will allow meaningful interpretation of clinical population PK data so that rational and robust guidelines may be generated to guide clinicians in optimising antibiotic, sedative and analgesic drug therapy during ECMO. The research methods described here are resource intensive and rely on extensive collaborations. Hopefully such an effort can be extended to comprehensively investigate many of the other complex issues in intensive care practice.

## Abbreviations

ECMO: Extracorporeal membrane oxygenation; ICU: Intensive care unit; PK: Pharmacokinetics; Vd: Volume of distribution; CL: Clearance; RASS: Richmond agitation sedation scale; HPLC: High performance liquid chromatography; LC-MS/MS: Liquid chromatography tandem mass spectrometry; RRT: Renal replacement therapy.

## Competing interests

The authors declared that they have no competing interest.

## Author’s contributions

KS designed the project and wrote the initial protocol. JAR, MTS, YLF and JFF provided further advice and input into the study design and the protocol. All authors read and approved the final manuscript.

## Pre-publication history

The pre-publication history for this paper can be accessed here:

http://www.biomedcentral.com/1471-2253/13/7/prepub
